# Supplementary material for: TRPV1 inhibition overcomes cisplatin resistance by blocking autophagy-mediated hyperactivation of EGFR signaling pathway
Source: Nat Commun. 2023 May 10;14:2691. doi: 10.1038/s41467-023-38318-7 (PMC10172196; doi:10.1038/s41467-023-38318-7)
Supplement: Supplementary file 1 — Description of Additional Supplementary Files [file 41467_2023_38318_MOESM1_ESM.pdf]

Title: Supplementary Data 1. The CESC patient's clinical characteristics.

Description: Clinical characteristics of CESC patients from The Cancer Genome Atlas (TCGA) data organized in the Firehose BROAD GDAC data repository (https://gdac.broadinstitute.org/).
