# Supplementary material for: TRPV1 inhibition overcomes cisplatin resistance by blocking autophagy-mediated hyperactivation of EGFR signaling pathway
Source: Nat Commun. 2023 May 10;14:2691. doi: 10.1038/s41467-023-38318-7 (PMC10172196; doi:10.1038/s41467-023-38318-7)
Supplement: Supplementary file 2 — Supplementary Information [file 41467_2023_38318_MOESM2_ESM.pdf]

supplementary Information for the manuscript entitled:

TRPV1 inhibition overcomes cisplatin resistance by blocking autophagy-mediated hyperactivation of EGFR signaling pathway

Se Jin Oh et al.

Supplementary Figures and Figure legends.

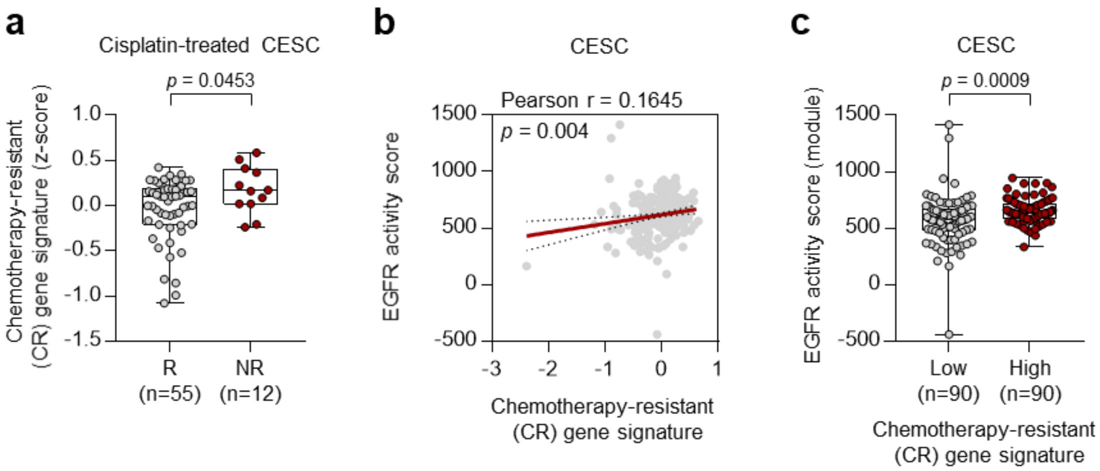

Supplementary Fig. 1. EGFR signaling is associated with chemotherapy resistance in cervical cancer patients. a Comparison of the level of chemotherapy-resistant (CR) gene signature in responder (R, *n* = 55) and non-responder (NR, *n* = 12) of cisplatin-treated CESC cohort. **b**. Correlation between expression level of CR gene signature and EGFR activity score in CESC cohort. **c**. Comparison of the level of EGFR activity scores in the CESC cohort with low levels (low, *n* = 90) and high levels (high, *n* = 90) of CR gene signature. The 30th and 70th percentiles were used as cutoffs values for the CR gene signature (CR gene signature^{high} > 70th; CR gene signature^{low} < 30th). In the box plots, the top and bottom edges of boxes indicate the first and third quartiles, respectively; the center lines indicate the medians; and the ends of whiskers indicate the maximum and minimum values, respectively. The *p*-values were determined by two-tailed Student's t-test (**a** and **c**) or spearman correlation (r) (**b**). The data represent the mean ± SD. Source data are provided as a Source Data file.

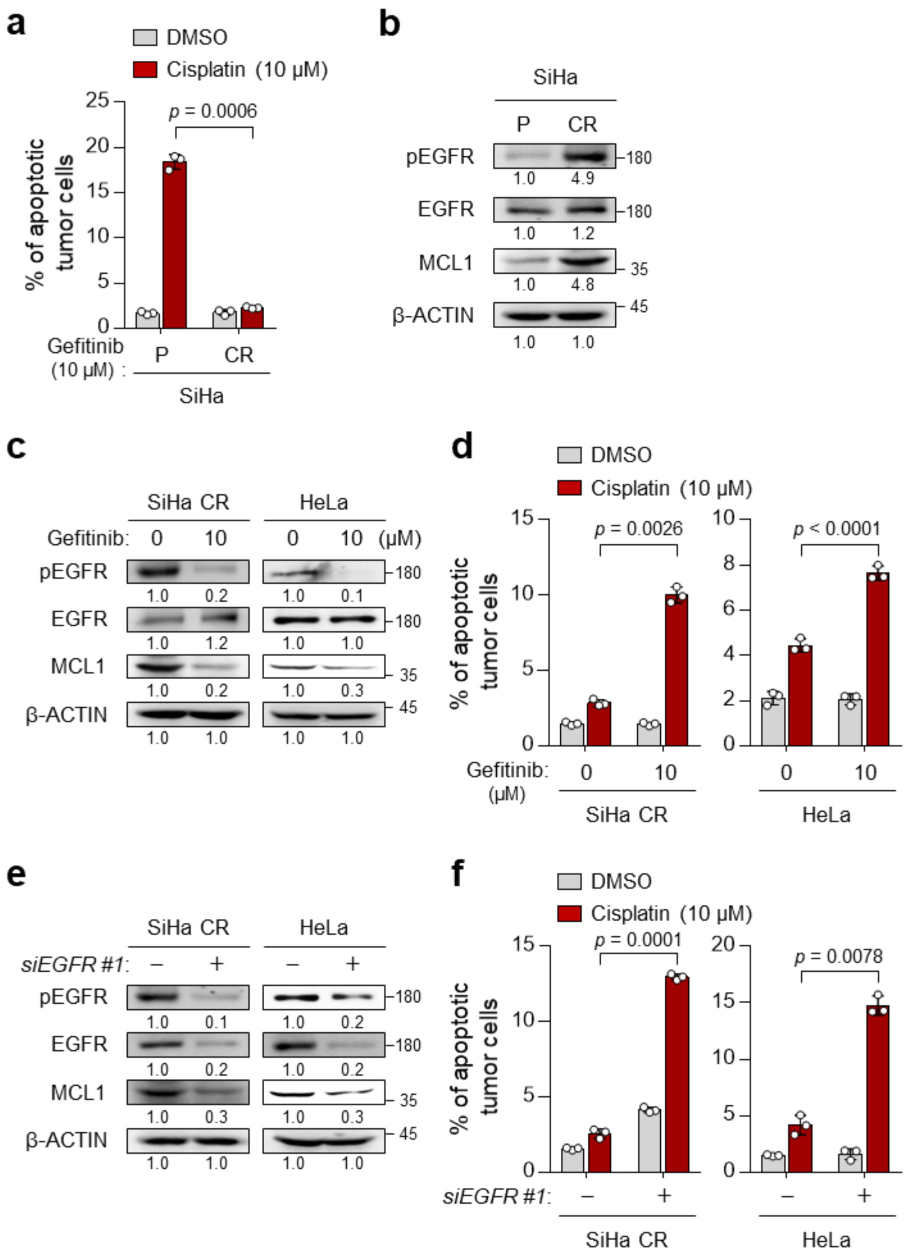

Supplementary Fig. 2. The phenotypes of cisplatin-resistant tumor cells are critically dependent on EGFR signaling activation. a Flow cytometry analysis of the frequency of apoptotic (active caspase 3^+) cells after treatment with or without cisplatin for 24 h. **b** The protein level of pEGFR, EGFR, and MCL1 expression were confirmed by western blots. **c** and **d** SiHa CR and HeLa cells were treated with gefitinib as the indicated dose. **e** and **f** SiHa CR and HeLa cells were transfected with siRNA targeting *GFP* or *EGFR*. **c** and **e** The protein levels of pEGFR, EGFR and MCL1 were determined by western blots. β-actin was used as an internal loading control. Numbers below the blot images indicate the expression as measured by fold-change. **d** and **f** Flow cytometry analysis of the frequency of apoptotic

(active caspase 3^+) cells after incubation with or without cisplatin for 24 h. All experiments were performed in triplicate. The *p*-values by two-way ANOVA are indicated. The data represent the mean ± SD. Source data are provided as a Source Data file.

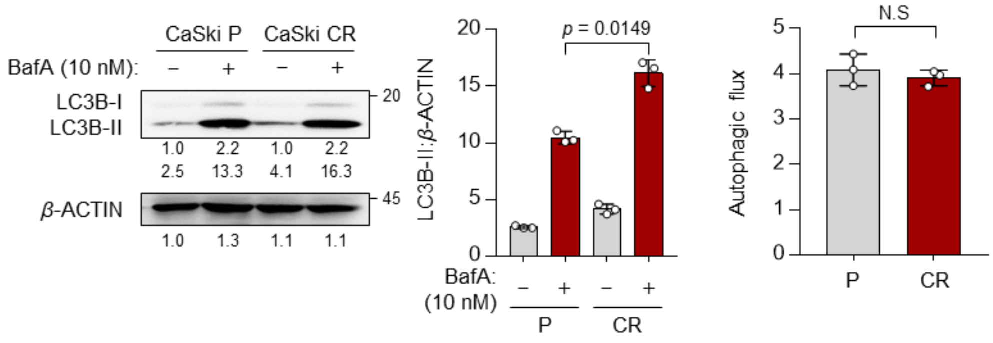

Supplementary Fig. 3. Autophagy flux is not altered in cisplatin-resistant tumor cells. CaSki P or CR cells were starved in medium supplemented with 0.1% FBS for 48 h and then incubated with or without BafA (10nM) for 12 h. The protein levels of LC3B were confirmed by western blots. β-actin was used as an internal loading control. Numbers below the blot images indicate the expression as measured by fold-change. The graphs represent quantification for absolute level of LC3B-II (left, ***P ≤ 0.001, by two-way ANOVA) and autophagic flux (right, p = N.S by 2-tailed Student`s t-test), respectively. Autophagic flux was calculated by dividing the value of LC3B-II in the presence of BafA by that absence BafA. All experiments were performed in triplicate. The *p*-values by two-way ANOVA (middle) or two-tailed Student’s t-test (right) are indicated. The data represent the mean ± SD. Source data are provided as a Source Data file. (NS, not significant)

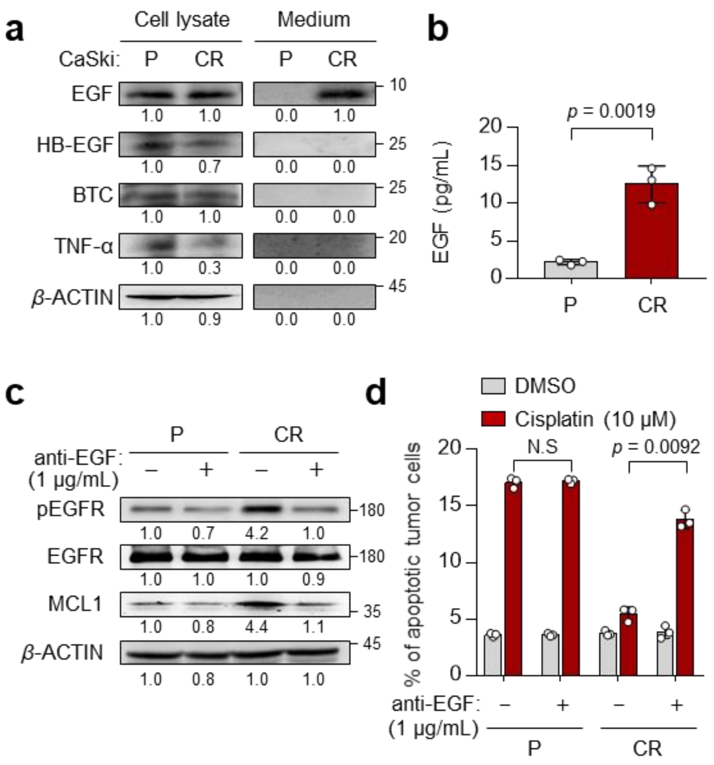

Supplementary Fig. 4. Secreted EGF plays crucial roles in cisplatin-resistant phenotypes in tumor cells. a The levels of internal EGF, HB-EGF, BTC, and TNF-α and secreted EGF, HB-EGF, BTC, TNF-α protein were determined by western blot analysis. **b** The amount of EGF secreted into the media was measured by ELISA. **c** and **d** CaSki P or CR cells were treated with IgG or anti-EGF. **c** The levels of pEGFR, EGFR, and MCL1 protein were confirmed by western blots. β-actin was used as an internal loading control. Numbers below the blot images indicate the expression as measured by fold-change. **d** The frequency of apoptotic (active caspase 3^+) cells were analyzed by flow cytometry. All experiments were performed in triplicate. The *p*-values by two-tailed Student's t-test (**b**) or two-way ANOVA (**d**) are indicated. The data represent the mean ± SD. Source data are provided as a Source Data file. (NS, not significant)

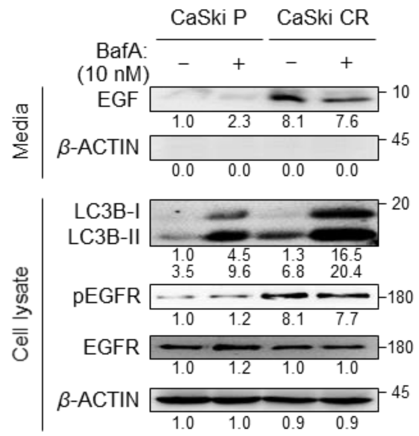

Supplementary Fig. 5. Increased EGF secretion in cisplatin-resistant tumor cells is not affected by treatment with BafA. In the cells treated with or without BafA, the protein levels of secreted EGF and internal LC3B, pEGFR, EGFR, and β-actin were determined by western blots. β-actin was used as an internal loading control. Numbers below the blot images indicate the expression as measured by fold-change. This experiment was performed in triplicate.

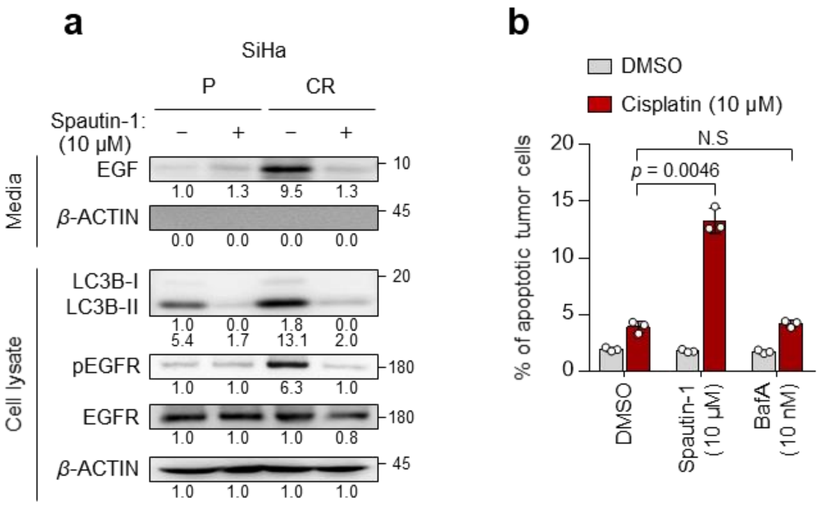

Supplementary Fig. 6. Autophagosome formation is crucial for EGF secretion and EGFR signaling-mediated cisplatin resistance in SiHa CR cells. a In the cells treated with or without spautin-1, the protein levels of secreted EGF and internal LC3B, pEGFR, EGFR, and β-actin were determined by western blots. β-actin was used as an internal loading control. Numbers below the blot images indicate the expression as measured by fold-change. **b** Flow cytometry analysis of the frequency of apoptotic (active caspase 3⁺) cells after incubation with or without cisplatin for 24 h. All experiments were performed in triplicate. The *p*-values by two-way ANOVA are indicated. The data represent the mean ± SD. Source data are provided as a Source Data file. (NS, not significant)

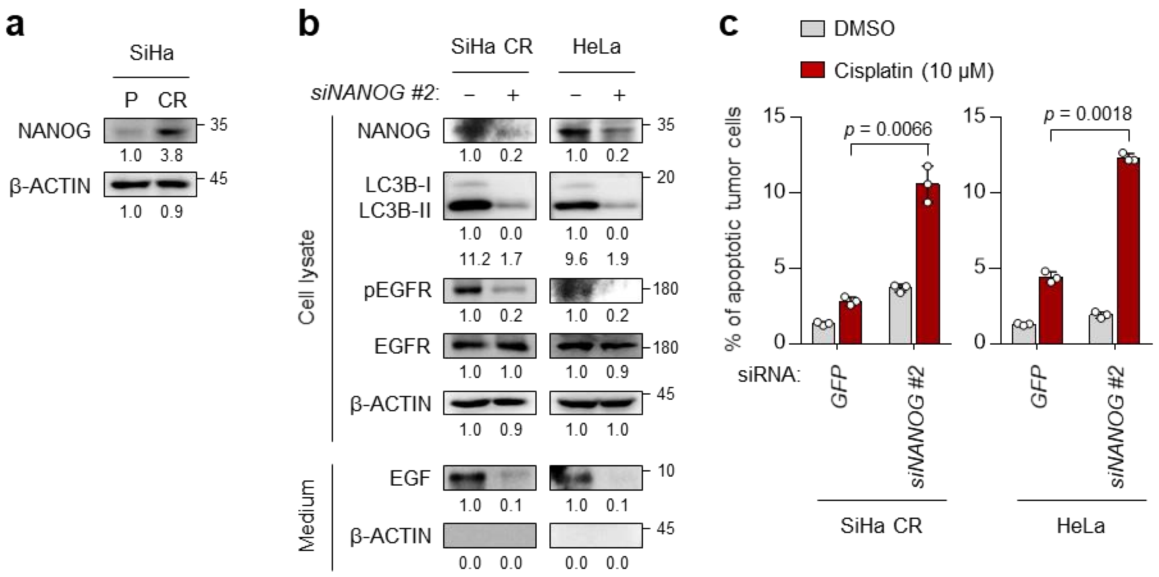

Supplementary Fig. 7. Knockdown of *NANOG* reduces cisplatin resistance by blocking autophagosome-mediated EGF secretion. a The protein levels of NANOG were determined by western blot. **b** and **c** SiHa CR or HeLa cells were transfected with siRNA targeting *GFP* or *NANOG*. **b** The levels of internal NANOG, LC3B, pEGFR, and EGFR and secreted EGF protein were confirmed by western blots. **c** Flow cytometry analysis of the frequency of apoptotic (active caspase 3^+) cells after incubation with or without cisplatin for 24 h. **a** and **b** β-actin was used as an internal loading control. Numbers below the blot images indicate the expression as measured by fold-change. All experiments were performed in triplicate. The *p*-values by two-way ANOVA are indicated. The data represent the mean ± SD. Source data are provided as a Source Data file.

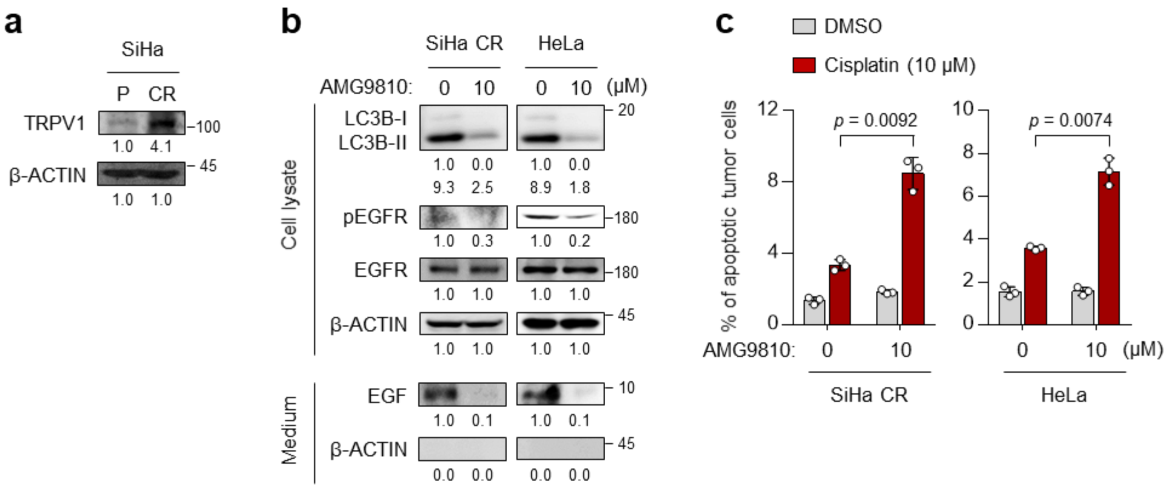

Supplementary Fig. 8. TRPV1 inhibition reduces cisplatin resistance by blocking autophagosome-mediated EGF secretion. a The protein levels of TRPV1 were determined by western blot. **b** and **c** SiHa CR or HeLa cells were treated with DMSO or AMG9810. **b** The levels of internal LC3B, pEGFR, and EGFR and secreted EGF protein were confirmed by western blots. **c** Flow cytometry analysis of the frequency of apoptotic (active caspase 3^+) cells after incubation with or without cisplatin for 24 h. **a** and **b** β-actin was used as an internal loading control. Numbers below the blot images indicate the expression as measured by fold-change. All experiments were performed in triplicate. The *p*-values by two-way ANOVA are indicated. The data represent the mean ± SD. Source data are provided as a Source Data file.

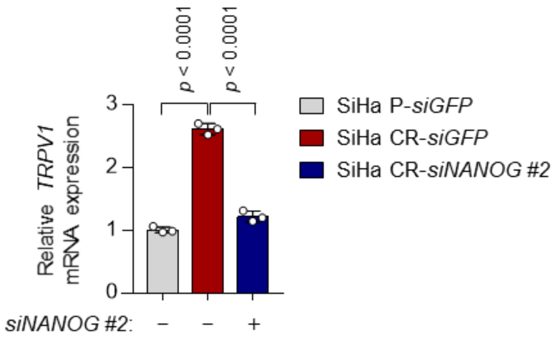

Supplementary Fig. 9. NANOG transcriptionally upregulates TRPV1 expression in SiHa cells. SiHa P and CR cells were transfected with siRNA targeting *GFP* or *NANOG*, as indicated. mRNA expression of *TRPV1* was analyzed by qRT-PCR. This experiment was performed in triplicate. The *p*-values by one-way ANOVA are indicated. The data represent the mean ± SD. Source data are provided as a Source Data file.

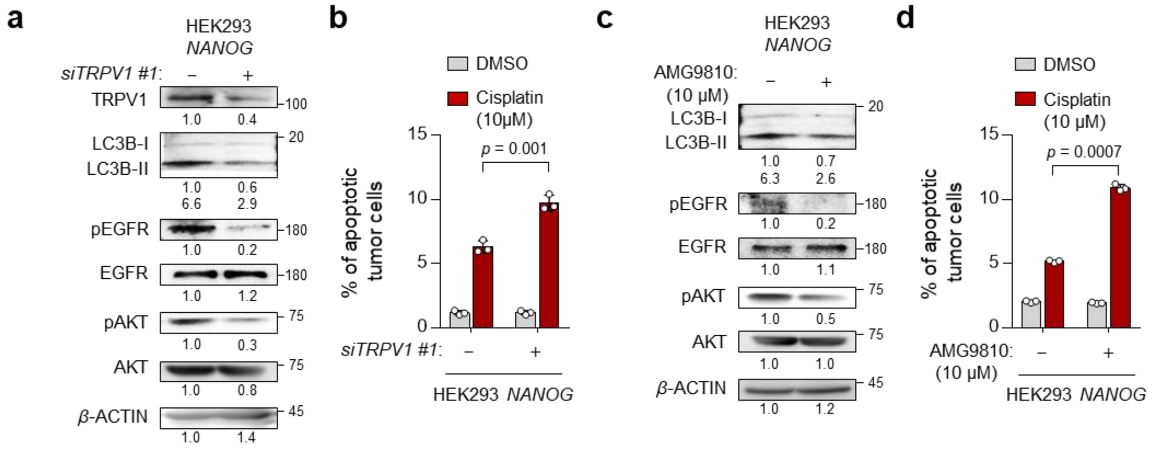

Supplementary Fig. 10. TRPV1 is required for NANOG-mediated refractory phenotypes. **a** and **b** HEK293 *NANOG* cells were transfected with siRNA targeting *GFP* or *TRPV1*. **c** and **d** HEK293 *NANOG* cells were treated with DMSO or AMG9810. **a** and **c** The protein levels of TRPV1, LC3B, pEGFR, EGFR, pAKT, and AKT were analyzed by western blots. β-actin was used as an internal loading control. Numbers below the blot images indicate the expression as measured by fold-change. **b** and **d** Flow cytometry analysis of the frequency of apoptotic (active caspase 3^+) cells after incubation with or without cisplatin for 24 h. All experiments were performed in triplicate. The *p*-values by two-way ANOVA are indicated. The data represent the mean ± SD. Source data are provided as a Source Data file.

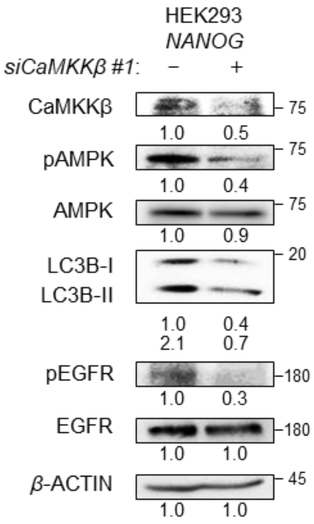

Supplementary Fig. 11. Ca^{2+}-AMPK pathway is required for NANOG-mediated EGFR activation. HEK293 NANOG cells were transfected siRNA targeting *GFP* or *CaMKKβ*. The levels of CaMKKβ, pAMPK, AMPK, LC3B, pEGFR, and EGFR were confirmed by western blot analysis. β-actin was used as an internal loading control. Numbers below the blot images indicate the expression as measured by fold-change. This experiment was performed in triplicate.

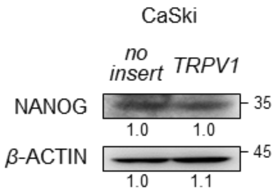

Supplementary Fig. 12. TRPV1 overexpression does not affects levels of NANOG. CaSki P cells were transfected with empty vector (*no insert*) or *TRPV1*. The levels of NANOG protein were confirmed by western blots. β-actin was used as an internal loading control. Numbers below the blot images indicate the expression as measured by fold-change. This experiment was performed in triplicate.

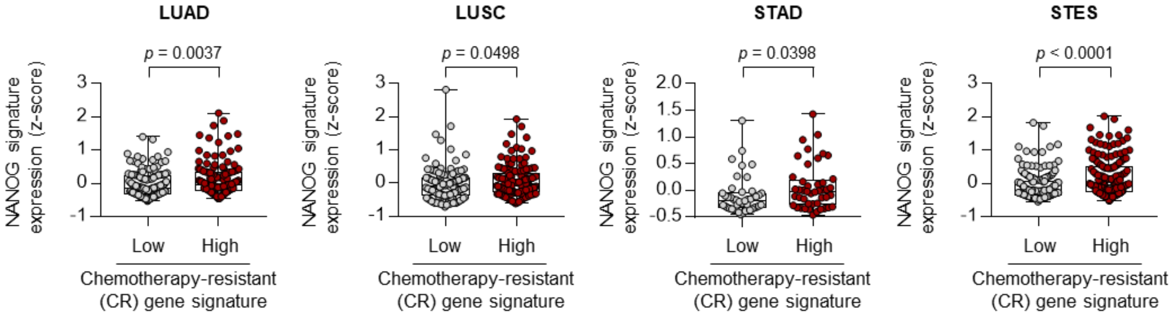

Supplementary Fig. 13. NANOG signature is upregulated in chemotherapy-resistant lung and gastric cancer patients. Comparisons of the expression levels of EGFR activity score in LUAD, LUSC, STAD, and STES cohort with low levels and high levels of chemotherapy-resistant gene signature. The 25th and 75th percentiles were used as cutoff thresholds. The number of patients analyzed for each cohort is as follows: LUAD (CR^{Low}, n = 144; CR^{high}, n = 144), LUSC (CR^{Low}, n = 138; CR^{high}, n = 138), STAD (CR^{Low}, n = 45; CR^{high}, n = 45), and STES (CR^{Low}, n = 161; CR^{high}, n = 161). The p-values were determined by unpaired, two-tailed Student's t-test. In the box plots, the top and bottom edges of the boxes indicate the first and third quartiles, respectively; the center lines indicate the medians, and the ends of the whiskers indicate the maximum and minimum values. Source data are provided as a Source Data file.

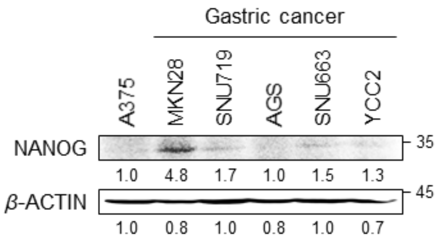

Supplementary Fig. 14. Analysis of NANOG expression in human gastric cancer cell lines. The levels of NANOG protein were determined by western blot analysis. β-actin was used as an internal loading control. Numbers below the blot images indicate the expression as measured by fold-change. This experiment was performed in triplicates.

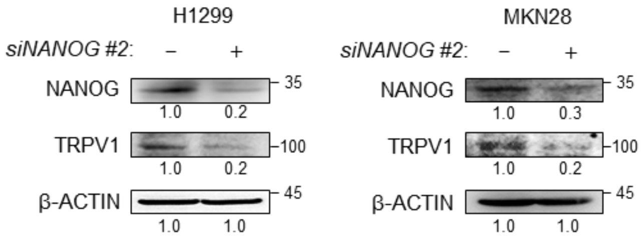

Supplementary Fig. 15. The NANOG-TRPV1 axis is conserved in NANOG^{high} lung and gastric cancer types. H1299 or MKN28 cells were transfected siRNA targeting *GFP* or *NANOG*. The protein levels of NANOG and TRPV1 were analyzed by western blots. β-actin was used as an internal loading control. Numbers below the blot images indicate the expression as measured by fold-change. This experiment was performed in triplicates.

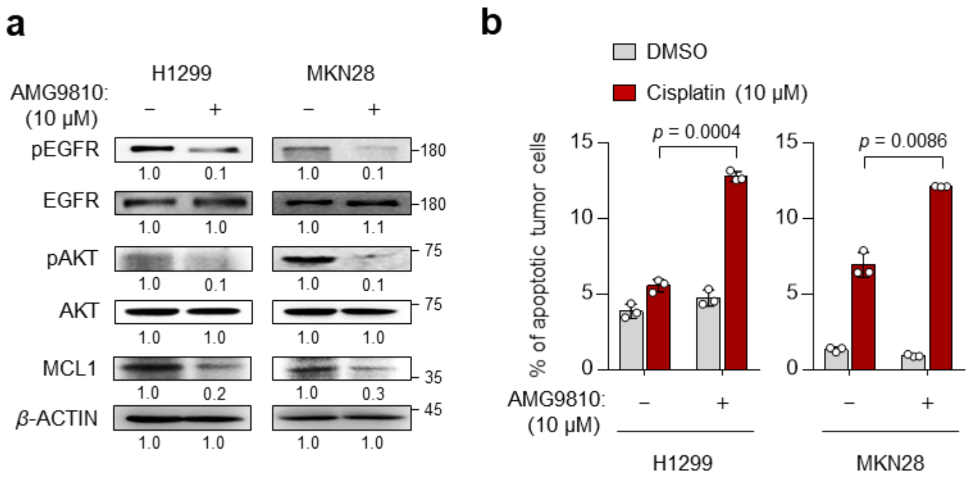

Supplementary Fig. 16. TRPV1 is a potential target for controlling NANOG^{high} tumor cells. a and **b** H1299 or MKN28 were treated with DMSO or AMG9810. a The protein levels of pEGFR, EGFR, pAKT, AKT, and MCL1 were confirmed by western blots. β-actin was used as an internal loading control. Numbers below the blot images indicate the expression as measured by fold-change. **b** Flow cytometry analysis of the frequency of apoptotic (active caspase 3^{+}) cells after incubation with or without cisplatin for 24 h. All experiments were performed in triplicates. The *p*-values by two-way ANOVA are indicated. The data represent the mean ±SD. Source data are provided as a Source Data file.

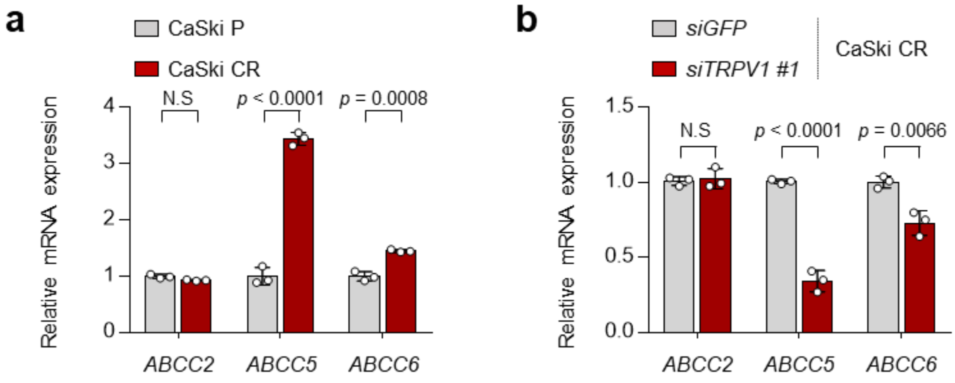

Supplementary Fig. 17. TRPV1 regulates expression of ABC transporter in cisplatin-resistant tumor cells. a The mRNA levels or indicated genes were measured by qRT-PCR in CaSki P or CR cells. **b** CaSki CR cells were transfected with siRNA targeting *GFP* or *TRPV1*. *ABCC2*, *ABCC5*, and *ABCC6* mRNA levels were analyzed by qRT-PCR. **a** and **b** The graphs represent three independent experiments performed in triplicate. The *p*-values by two-tailed Student's t-test are indicated. The data represent the mean ± SD. Source data are provided as a Source Data file. (NS, not significant)

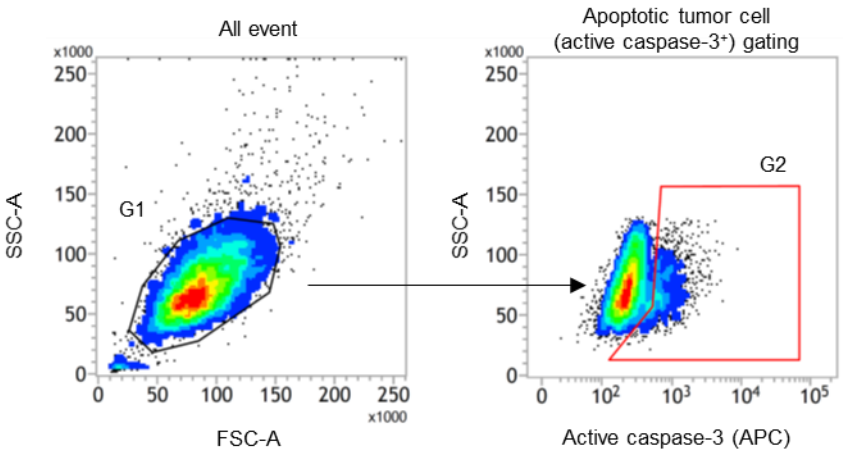

Supplementary Fig. 18. Gating strategy for identification of apoptotic tumor cells by cisplatin treatment. Representative flow cytometry analysis showing the gating strategy. Population was initially gated on the basis of the forward side scatter characteristics and debris was eliminated. After then, the active caspase-3$^+$ population was measured in the active capase-3 (APC).

Supplementary Fig. 19. Uncropped blot images

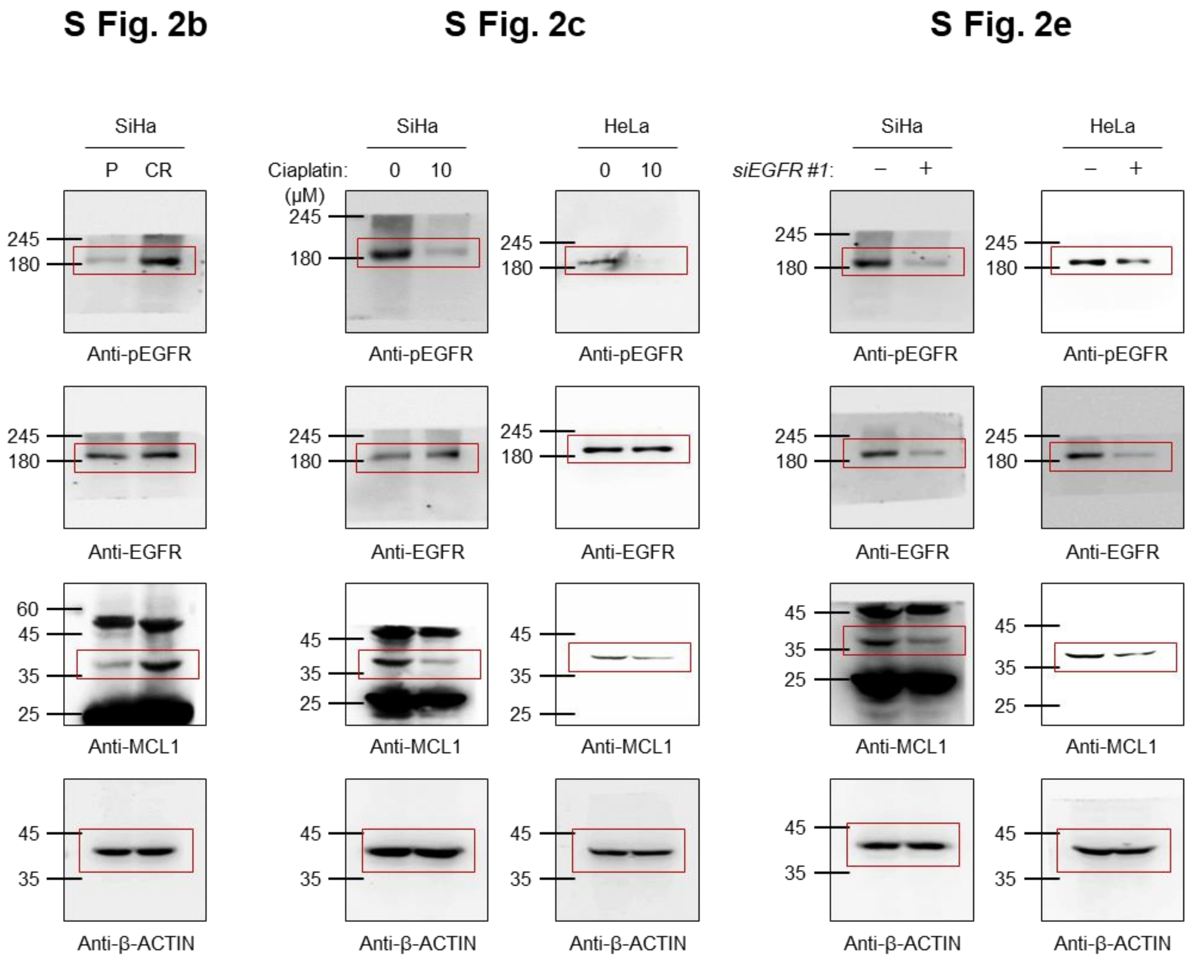

Supplementary Fig. 19. Uncropped blot images (continued)

S Fig. 3

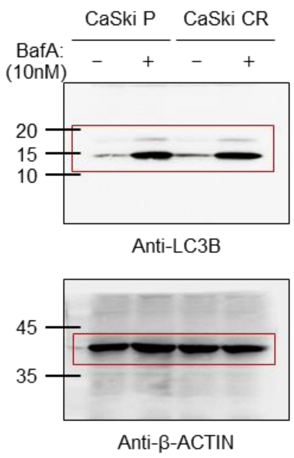

S Fig. 4a

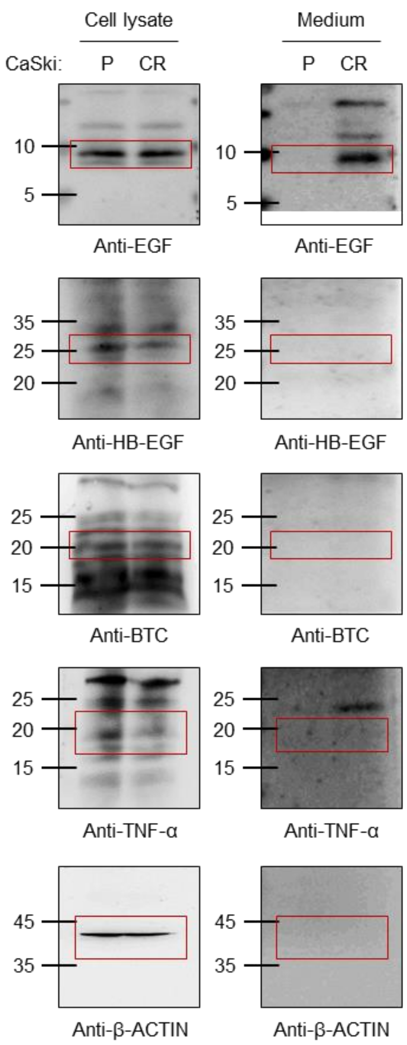

S Fig. 4c

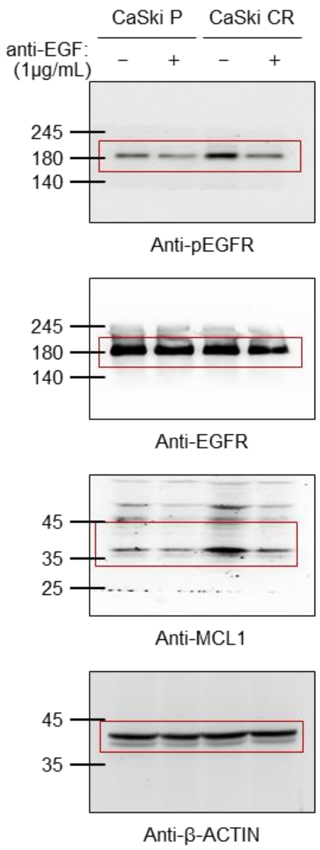

Supplementary Fig. 19. Uncropped blot images (continued)

S Fig. 5

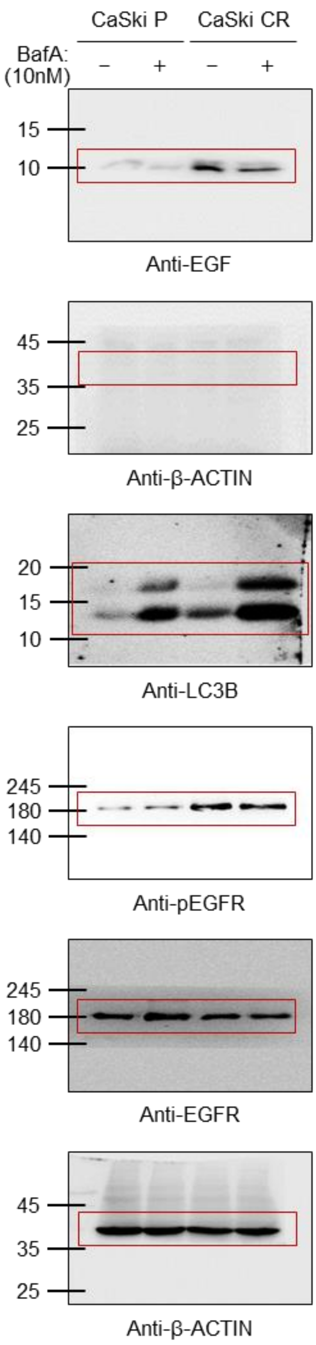

S Fig. 6a

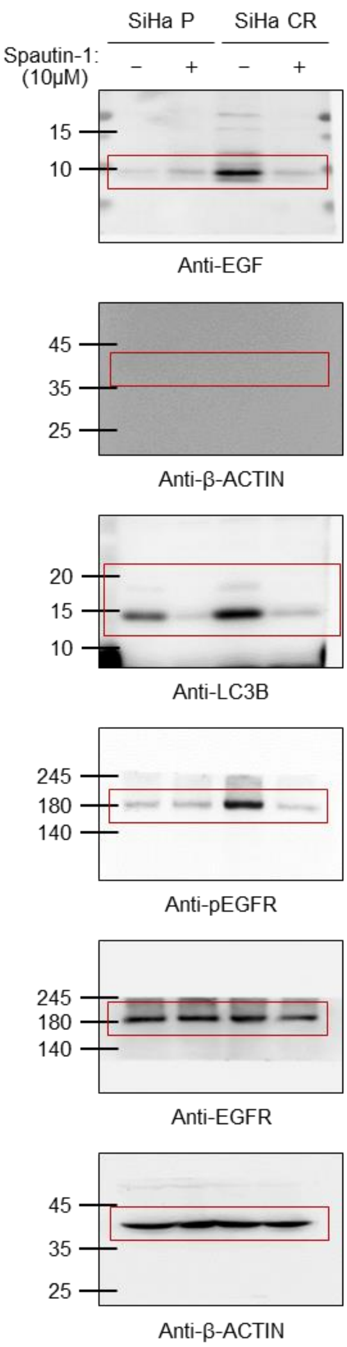

Supplementary Fig. 19. Uncropped blot images (continued)

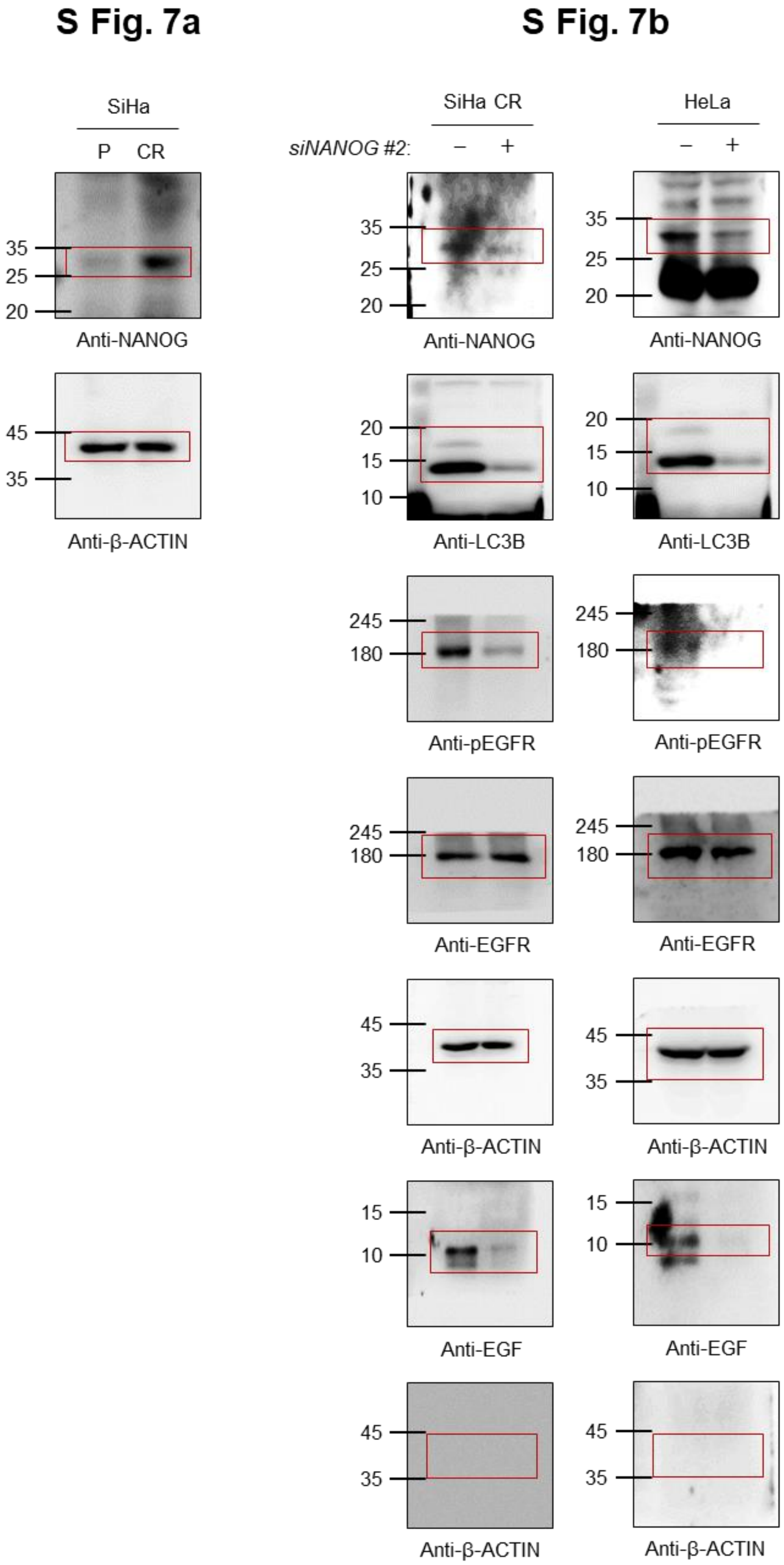

Supplementary Fig. 19. Uncropped blot images (continued)

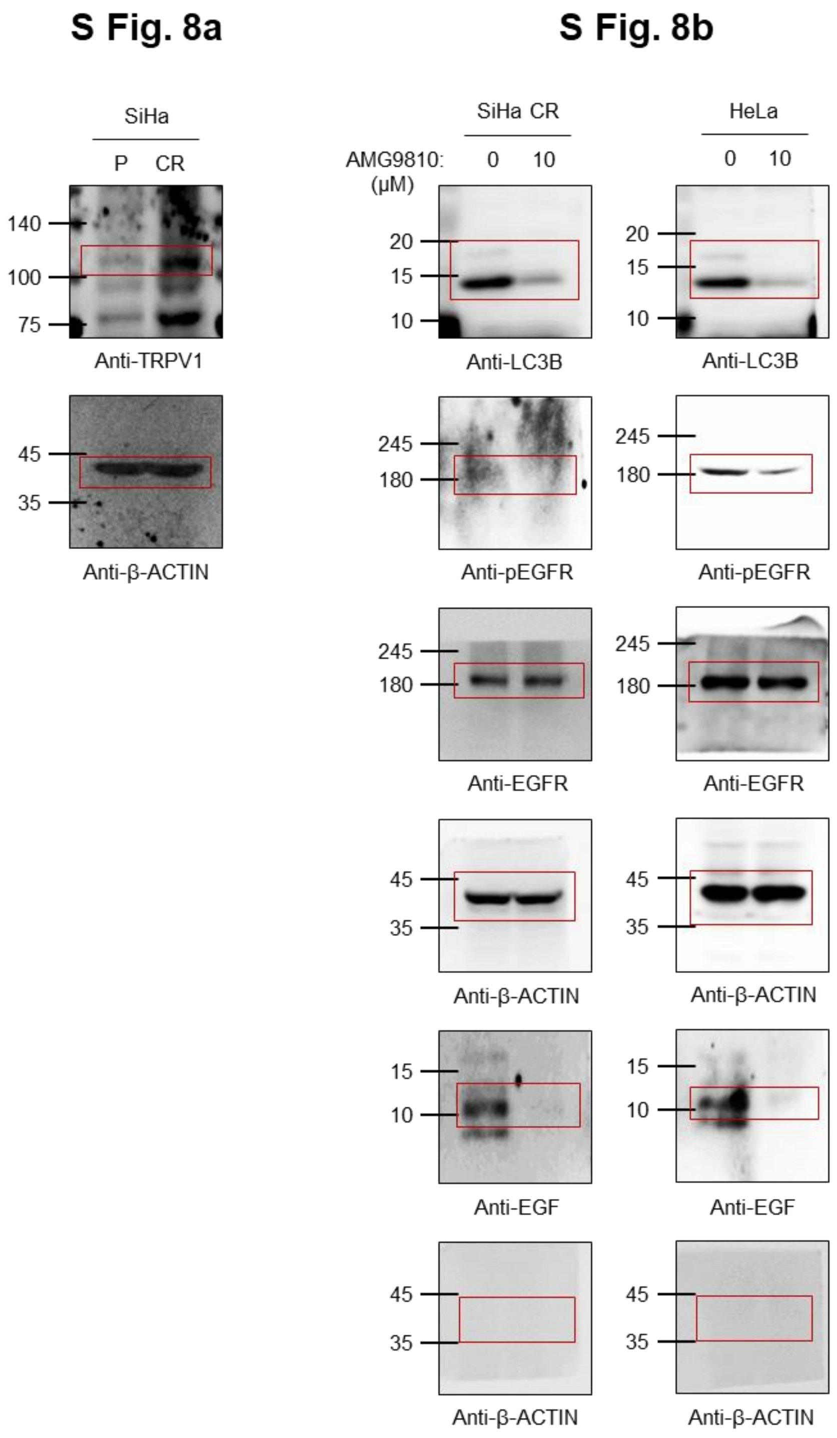

Supplementary Fig. 19. Uncropped blot images (continued)

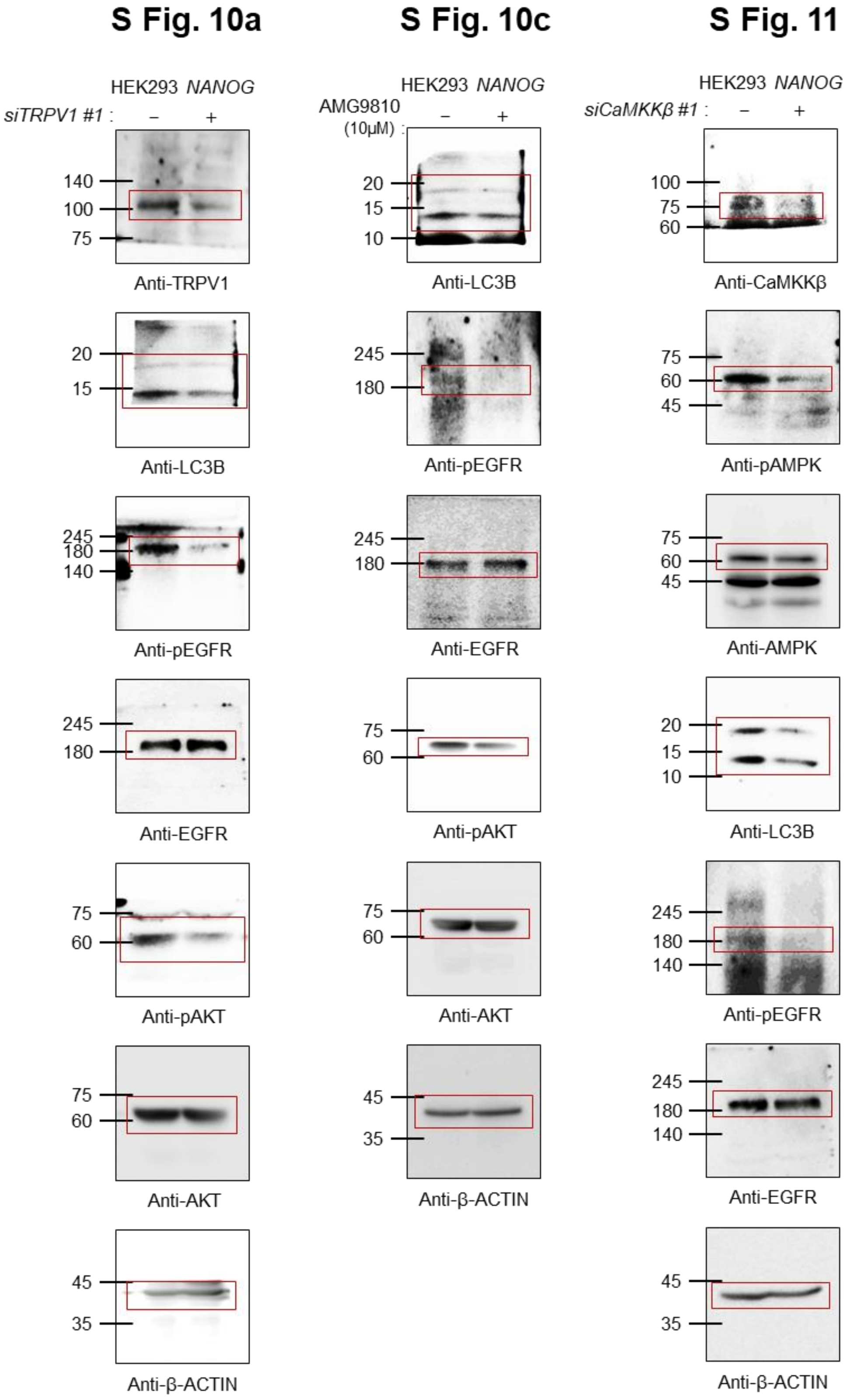

Supplementary Fig. 19. Uncropped blot images (continued)

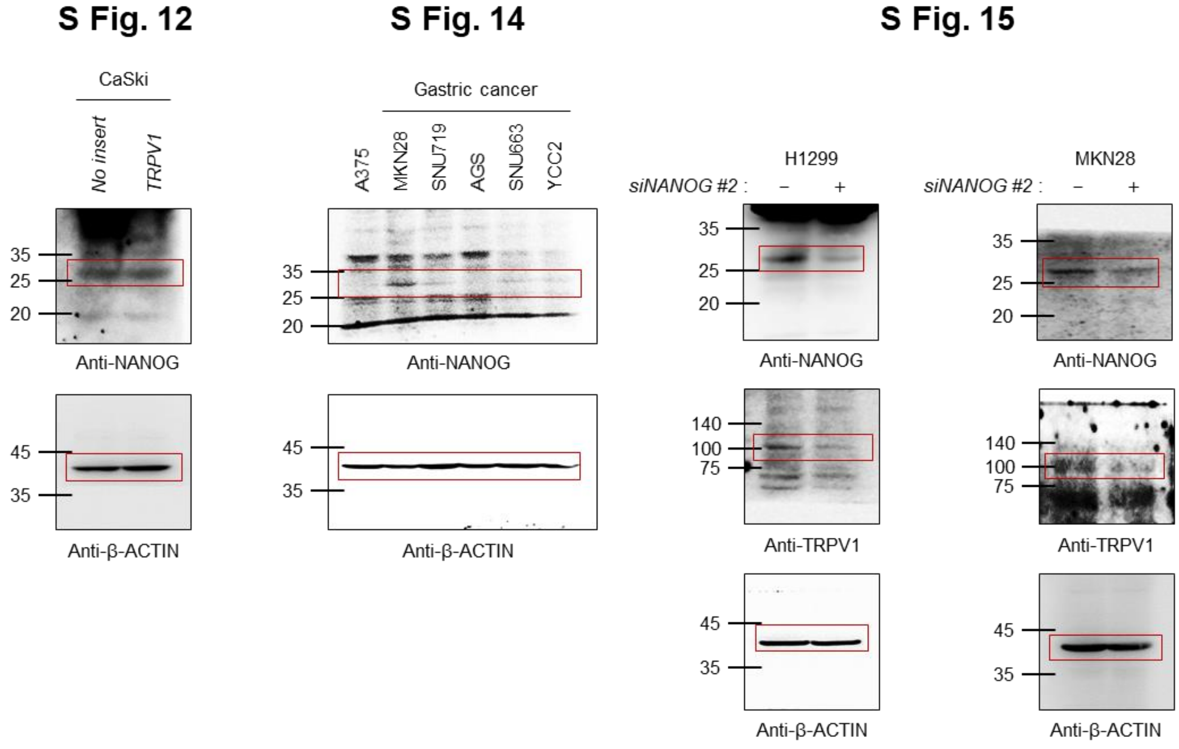

Supplementary Fig. 19. Uncropped blot images (continued)

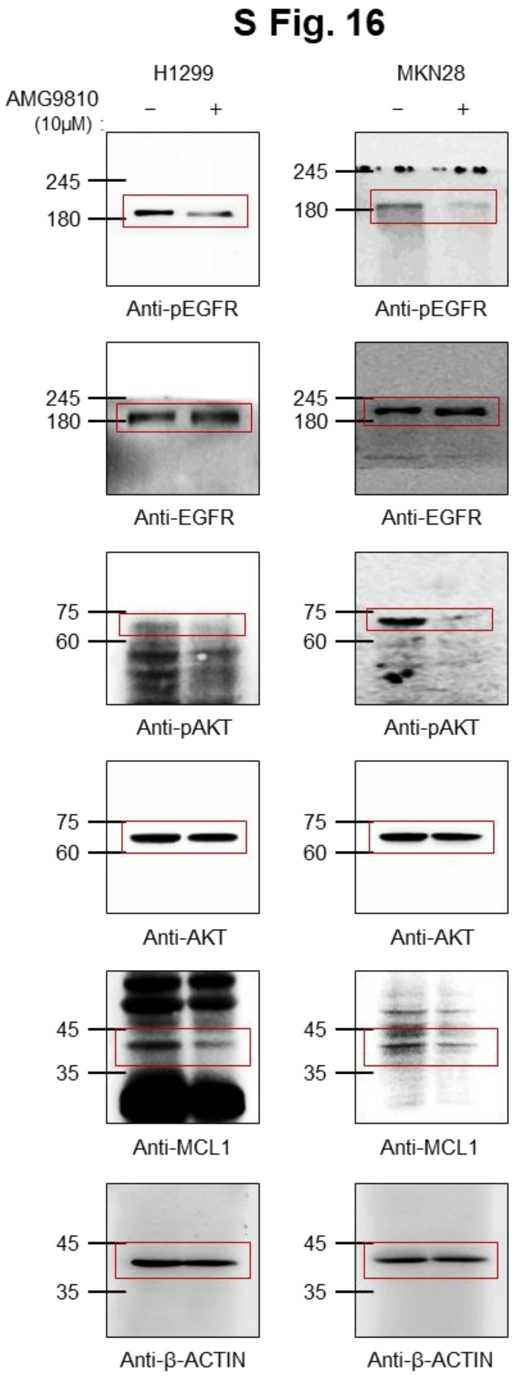

Supplementary Table 1. Clinicopathological characteristics patients with cervical cancer.

	No.	Chemoradiation response		*p* value
		Good<br>No. (%)	Bad<br>No. (%)	
**Age**				0.845
≤ 50	42	28 (52.8)	14 (58.3)	
> 50	35	25 (47.2)	10 (41.7)	
**FIGO stage**				<0.001
I	40	36 (67.9)	4 (16.7)	
II	32	17 (32.1)	15 (62.5)	
IV	5	0	5 (20.8)	
**Tumor grade**				0.293
Well	2	1 (1.9)	1 (4.2)	
Moderate	49	37 (69.8)	12 (50.0)	
Poor	25	14 (26.4)	11 (45.8)	
Unknown	1	1 (1.9)	0	
**Cell type**				0.316
SCC	60	40 (75.4)	20 (83.4)	
AD	13	11 (20.8)	2 (8.3)	
Others	4	2 (3.8)	2 (8.3)	
**Tumor size**				1.000
≤ 4 cm	43	30 (56.6)	13 (54.2)	
> 4 cm	34	23 (43.4)	11 (45.8)	
**Lymphnode metastasis**				0.111
Absent	36	29 (54.7)	7 (29.2)	
Present	25	15 (28.3)	10 (41.7)	
Unknown	16	9 (17.0)	7 (29.2)	
**Lymphovascular invasion**				0.014
Absent	20	18 (34.0)	2 (8.2)	
Present	36	25 (47.2)	11 (45.8)	
Unknown	21	10 (18.8)	11 (45.8)	
**SCC antigen**				
Negative	40	31 (58.5)	9 (37.5)	
Positive	27	15 (28.3)	12 (50.0)	
Unknown	10	7 (13.2)	3 (12.5)	
**HPV**				0.141
Negative	2	2 (3.8)	0	
Positive	42	32 (60.4)	10 (41.7)	
Unknown	33	19 (35.8)	14 (58.3)	
**Recur**				<0.001
Absent	45	45 (84.9)	0	
Present	32	8 (15.1)	24 (100.0)	
**Status**				<0.001
Alive	60	49 (92.5)	11 (45.8)	
Expire	17	4 (7.5)	13 (54.2)	

Statistical analyses were performed using the SPSS Statistics for Windows, version 21 (IBM Corp, Armonk, NY, USA). Differences in clinicopathological features between good and bad of chemoradiation

response were analyzed using Chi-square or Fisher's exact test for categorical variables. FIGO, International Federation of Gynecology and Obstetrics; SSC, squamous cell carcinoma; AD, adenocarcinoma; HPV, human papillomavirus.
