# Supplementary material for: TRPV1 inhibition overcomes cisplatin resistance by blocking autophagy-mediated hyperactivation of EGFR signaling pathway
Source: Nat Commun. 2023 May 10;14:2691. doi: 10.1038/s41467-023-38318-7 (PMC10172196; doi:10.1038/s41467-023-38318-7)
Supplement: Supplementary file 5 — Reporting Summary [file 41467_2023_38318_MOESM5_ESM.pdf]

nature portfolio

Corresponding author(s): Tae Woo Kim
Last updated by author(s): Mar 7, 2023

Reporting Summary

Nature Portfolio wishes to improve the reproducibility of the work that we publish. This form provides structure for consistency and transparency in reporting. For further information on Nature Portfolio policies, see our Editorial Policies and the Editorial Policy Checklist.

Statistics

For all statistical analyses, confirm that the following items are present in the figure legend, table legend, main text, or Methods section.

n/a	Confirmed	
☐	☒	The exact sample size (*n*) for each experimental group/condition, given as a discrete number and unit of measurement
☐	☒	A statement on whether measurements were taken from distinct samples or whether the same sample was measured repeatedly
☐	☒	The statistical test(s) used AND whether they are one- or two-sided<br>*Only common tests should be described solely by name; describe more complex techniques in the Methods section.*
☐	☒	A description of all covariates tested
☐	☒	A description of any assumptions or corrections, such as tests of normality and adjustment for multiple comparisons
☐	☒	A full description of the statistical parameters including central tendency (e.g. means) or other basic estimates (e.g. regression coefficient) AND variation (e.g. standard deviation) or associated estimates of uncertainty (e.g. confidence intervals)
☐	☒	For null hypothesis testing, the test statistic (e.g. *F*, *t*, *r*) with confidence intervals, effect sizes, degrees of freedom and *P* value noted<br>*Give P values as exact values whenever suitable.*
☒	☐	For Bayesian analysis, information on the choice of priors and Markov chain Monte Carlo settings
☒	☐	For hierarchical and complex designs, identification of the appropriate level for tests and full reporting of outcomes
☐	☒	Estimates of effect sizes (e.g. Cohen's *d*, Pearson's *r*), indicating how they were calculated

Our web collection on statistics for biologists contains articles on many of the points above.

Software and code

Policy information about availability of computer code

Data collection	No software was used for data collection
Data analysis	GraphPad Prism Ver. 7.0 (GraphPad Software, Inc., San Deigo, CA, USA), Seurat (v4.0.6)`s `AddModuleScore', Image J 1.53g, FACSuite software version 1.0.5.

For manuscripts utilizing custom algorithms or software that are central to the research but not yet described in published literature, software must be made available to editors and reviewers. We strongly encourage code deposition in a community repository (e.g. GitHub). See the Nature Portfolio guidelines for submitting code & software for further information.

Data

Policy information about availability of data

All manuscripts must include a data availability statement. This statement should provide the following information, where applicable:

- Accession codes, unique identifiers, or web links for publicly available datasets
- A description of any restrictions on data availability
- For clinical datasets or third party data, please ensure that the statement adheres to our policy

RNA-seq dataset and clinical data from various cancer types, such as CESC, LUAD, LUSC, STAD, or STES, are publicly available in the Firehose BROAD GDAC data repository (https://gdac.broadinstitute.org/). The source data underlying Fig. 1-8 and Supplementary Fig. 1-17 are provided in Source data file with this paper. The gating strategy is provided in Supplementary Fig. 18. All raw images for the immunoblots are provided in Supplementary Fig. 19. The remaining data are available within the article, Supplementary Information or Source Data file.

Human research participants

Policy information about studies involving human research participants and Sex and Gender in Research.

Reporting on sex and gender	The study subjects were compromised of 77 surgically resected primary cervical cancer patients (53 chemoradiosensitive and 24 chemoradioresistant).
Population characteristics	The patient`s clinicopathological characteristics are summarized in Supplementary Table 1.
Recruitment	The study collected tissue samples from patients who underwent either type 3 radical hysterectomy with pelvic lymph node (LN) dissection or conization at Gangnam Severance Hospital between 1996 and 2010. The gynecology pathologists confirmed that all specimens included in the study were adequate and provided relevant clinicopathological information, such as age, FIGO stage, tumor grade, cell type, tumor size, LN metastasis, involvement of lymphovascular space invasion (LVIS), survival time, survival status, and response to concurrent chemoradiation therapy (CCRT). However, due to the limited number of samples with available chemoradiation response results, only 77 were used for analysis. This small sample size can be considered a limitation of the study.
Ethics oversight	This study received approval from the Institutional Review Board of Gangnam Severance Hospital (Seoul, KOR), and informed consent was obtained from each patient after providing a detailed explanation of the study procedures, including the risks and benefits of participating. Once the patient had a full understanding of the study, they provided written informed consent to participate. All procedures were conducted in accordance with the guidelines of the Declaration of Helsinki.

Note that full information on the approval of the study protocol must also be provided in the manuscript.

Field-specific reporting

Please select the one below that is the best fit for your research. If you are not sure, read the appropriate sections before making your selection.

☒ Life sciences ☐ Behavioural & social sciences ☐ Ecological, evolutionary & environmental sciences

For a reference copy of the document with all sections, see nature.com/documents/nr-reporting-summary-flat.pdf

Life sciences study design

All studies must disclose on these points even when the disclosure is negative.

Sample size	Mice number (n = 10) were determined by in vivo experience study to monitor and calculate percentage of mice survival of each experimental group.
Data exclusions	No data were excluded.
Replication	All data are representative of at least 3 separate experiments. All attempt at replication were successful
Randomization	For in vivo treatment experiments, tumor-bearing mice were subjected to caliper measurements. Animals with comparable tumor sizes were randomized into treatment groups; this prevented outcomes from being influenced by initial differences in tumor burden. For in vitro cell experiments, all cells in each experiment were from the same parental cells.
Blinding	To give different treatments to different experimental groups, the investigators were not blinded in Xenografts study. Analyses in the animal experiments as the results reported were based on measurements acquired. For RNA analyses using qRT-PCR and protein analyses by immunoblotting were not performed blind to load samples by order. Almost investigators were not blinded because the experiments we performed in this study was not applicable for blinding.

Reporting for specific materials, systems and methods

We require information from authors about some types of materials, experimental systems and methods used in many studies. Here, indicate whether each material, system or method listed is relevant to your study. If you are not sure if a list item applies to your research, read the appropriate section before selecting a response.

Materials & experimental systems

n/a	Involved in the study
☐	☒ Antibodies
☐	☒ Eukaryotic cell lines
☒	☐ Palaeontology and archaeology
☐	☒ Animals and other organisms
☒	☐ Clinical data
☒	☐ Dual use research of concern

Methods

n/a	Involved in the study
☒	☐ ChIP-seq
☐	☒ Flow cytometry
☒	☐ MRI-based neuroimaging

Antibodies

Antibodies used	The antibodies used in this study were anti-pEGFR (1:3000; Cell signaling, 3777S), anti-EGFR (1:3000; Cell signaling, 4267S), anti-MCL1 (1:3000; Santa Cruz Biotechnology, sc-819), anti-ATG7 (1:3000; Cell signaling, 8558S), anti-LC3B (1:3000; Cell signaling, 2775S), anti-EGF (1:1000; Abcam, ab206106), anti-NANOG (1:3000; Bethyl Laboratories, A300-379A), anti-TRPV1 (1:3000; Abcam, ab6166, immunoblot), anti-TRPV1 (1:1000; Alomone, ACC-030, IHC), anti-FLAG (1:5000; Medical & Biological Laboratories, M185-3L), anti-pAKT (1:3000; Cell signaling, 9271), anti-AKT1 (1:3000; Cell signaling, 9272), anti-β-actin (1:5000; Medical & Biological Laboratories, M177-3), anti-rabbit IgG-HRP (1:5000; Enzo, ADI-SAB-300-J), anti-mouse IgG-HRP (1:5000; Enzo, ADI-SAB-100-J), and anti-active caspase-3 (1:500; BD Biosciences, 560626).
Validation	Validation statements for antibodies can be found on their corresponding manufacturer websites. Validation in our own samples has been confirmed by Western blot detection of bands at the predicted size. anti-pEGFR (Cell signaling, 3777S), anti-EGFR (Cell signaling, 4267S), anti-MCL1 (Santa Cruz Biotechnology, sc-819), anti-ATG7 (Cell signaling, 8558S), anti-LC3B (Cell signaling, 2775S), anti-EGF (Abcam, ab206106), anti-NANOG (Bethyl Laboratories, A300-379A), anti-TRPV1 (Abcam, ab6166), anti-FLAG (Medical & Biological Laboratories, M185-3L), anti-pAKT (Cell signaling, 9271), anti-AKT1 (Cell signaling, 9272), anti-β-actin (Medical & Biological Laboratories, M177-3), anti-rabbit IgG-HRP (Enzo, ADI-SAB-300-J), and anti-mouse IgG-HRP (Enzo, ADI-SAB-100-J) were used for immunoblot analysis. Active caspase-3 (BD Biosciences, 560626) antibody was used for flow cytometry work. TRPV1 (Alomone, ACC-030) antibody was validated for IHC analysis.

Eukaryotic cell lines

Policy information about cell lines and Sex and Gender in Research

Cell line source(s)	CaSki (ATCC, CRL-1550), HEK293 (ATCC, CRL-1573), HeLa (ATCC, CCL-2), H1299 (ATCC, CRL-5803), SiHa (KCLB, 30035), SNU719 (KCLB, 00719), AGS (KCLB, 21739), SNU668 (KCLB, 00668), and MKN28 (JCRB Cell Bank, 0253) cell lines were purchased from the American Type Culture Collection (ATCC, Manassas, VA, USA), the Korean Cell Line Bank (KCLB, Seoul, KOR) or the Japanese Collection of Research Bioresources Cell Bank (JCRB Cell Bank, Osaka, JPN). YCC2 were obtained from Cancer Metastasis Research Center (CMRC, Yonsei University College of Medicine, Seoul, KOR). All cell lines were obtained between 2010 and 2022.<br>Generation of the CaSki CR cell line is described in Experimental & Molecular Medicine (2017) 49, e374.<br>Details about generation of the SiHa CR cell line are provided in the Supplementary information of the manuscript.<br>Generation of CaSki NANOG or HEK293 NANOG cell lines are described in Cancer Res (2017) 77. 5039-5053.
Authentication	The identities of cell lines were confirmed by short tandem repeat (STR) profiling by IDEXX Laboratories Inc. and used within 6 months for testing.
Mycoplasma contamination	All cell lines were tested for mycoplasma using Mycoplasma Detection Kit (Thermo Fisher Scientific, San Jose, CA, USA) and negative for mycoplasma contamination.
Commonly misidentified lines<br>(See ICLAC register)	No misidentified cell line was used in this study.

Animals and other research organisms

Policy information about studies involving animals; ARRIVE guidelines recommended for reporting animal research, and Sex and Gender in Research

Laboratory animals	Six- to eight-week-old female NOD-SCID mice were used in this project. All mice were maintained in SPF1 (specific pathogen free-1) condition and cared by Korea University Institutional Animal care center policy.

Wild animals	This study did not involve wild animals.
Reporting on sex	Because this study mainly targets cervical cancer with cisplatin resistance, only female mice were used for the experiment.
Field-collected samples	The study did not involve samples collected from the field
Ethics oversight	All mice were maintained and handled under the protocol approved by the Korea University Institutional Animal Care and Use Committee (KOREA-2021-0049). All animal procedures were performed in accordance with recommendations for the proper use and care of laboratory animals

Note that full information on the approval of the study protocol must also be provided in the manuscript.

Flow Cytometry

Plots

Confirm that:

- [x] The axis labels state the marker and fluorochrome used (e.g. CD4-FITC).
- [x] The axis scales are clearly visible. Include numbers along axes only for bottom left plot of group (a 'group' is an analysis of identical markers).
- [x] All plots are contour plots with outliers or pseudocolor plots.
- [x] A numerical value for number of cells or percentage (with statistics) is provided.

Methodology

Sample preparation	Tumor cells were treated with indicated agents in medium supplemented with 0.1% fetal bovine serum (FBS) for 24 h at 37℃. The cells were stained for active caspase-3 (BD Biosciences, Franklin Lakes, NJ, USA, 51-98955X) as an indicator of apoptosis and examine by flow cytometry
Instrument	FACSVerse flowcytometer (BD Biosciences, Cat no. #651154, year 2014)
Software	Data analysis was performed in BD FACSuite software.
Cell population abundance	Purity was determined by running flow cytometry of the sorted population.
Gating strategy	Tumor cells were first gated to exclude cell debris and aggregates based on FSC/SSC. Then cells stained with isotype control were used to determine the boundary between "negative" and "positive" cells; this boundary was used to identify positive cells in samples stained with the specific antibody.

- [x] Tick this box to confirm that a figure exemplifying the gating strategy is provided in the Supplementary Information.
